# Proline dehydrogenase, a rate-limiting catabolic enzyme, affecting the growth and pathogenicity of *Toxoplasma gondii* tachyzoites by regulating the proline metabolism and mitochondrial function of the parasite

**DOI:** 10.1186/s13071-025-06966-x

**Published:** 2025-07-29

**Authors:** Xiao-Ling Geng, Jing-Yu Li, Huan-Yu Xu, Jiang-Ping Wu, De-Liang Tao, Jin-Ming Chen, Ying-Ying Fan, Xin Yang, Jun-Ke Song, Guang-Hui Zhao

**Affiliations:** Department of Parasitology, College of Veterinary Medicine, Northwest A&F University, Yangling, 712100 China

**Keywords:** *Toxoplasma gondii*, *Tgprodh*, Growth, Proline metabolism, Mitochondrial function

## Abstract

**Background:**

The pathogenicity of *Toxoplasma gondii* is closely associated with its intracellular lytic cycle in host cells. Currently, the mechanisms by which *T. gondii* completes the lytic cycle remain unclear. The proline metabolism has been reported to be crucial for intracellular growth of pathogens by providing energy and nutrients. However, it remains unclear whether the intracellular growth and pathogenicity of *T. gondii* are related to proline metabolism.

**Methods:**

The gene-edited strains of proline dehydrogenase (*Tgprodh*) were constructed by using clustered regularly interspaced short palindromic repeats/CRISPR-associated protein 9 (CRISPR–Cas9) technology. The effects of the *Tgprodh* gene on the growth in vitro and pathogenicity in vivo of the tachyzoites for *T. gondii* were studied through proliferation, plaque, invasion, egress and virulence assays. The effects of the *Tgprodh* gene on mitochondrial function were studied by using reactive oxygen species (ROS), mitochondrial membrane potential (∆Ψm), adenosine triphosphate (ATP) assay kits, mitochondrial DNA (mtDNA) copy numbers, transmission electron microscopy (TEM) analysis, and reverse transcriptase quantitative polymerase chain reaction (RT-qPCR). The effects of the *Tgprodh* gene on proline metabolism were studied by using l-proline (L-Pro), l-glutamic acid (L-Glu), l-glutamine (L-Gln) assay kits, and RT-qPCR.

**Results:**

TgPRODH, the first rate-limiting enzyme in proline metabolism, was identified to be encoded by *T. gondii* and localized in the cytoplasm of *T. gondii*. Deletion of the *Tgprodh* gene resulted in significant growth inhibition in vitro and reduced pathogenicity in vivo of *T. gondii*. Further study found that deletion of the *Tgprodh* gene caused damage to the mitochondrial morphology, decreased membrane potential, mtDNA copy numbers, and the production of ATP and ROS. The expression of genes for maintaining mitochondrial integrity was downregulated in the *Tgprodh*-knockout strain of *T. gondii*, while complementation of the *Tgprodh* gene restored these defects in this parasite. Meantime, the deletion of the *Tgprodh* gene resulted in the accumulation of proline, reduced the contents of glutamate and glutamine, and affected the expression of genes related to proline catabolism in *T. gondii*.

**Conclusions:**

The present study found the significance of the *Tgprodh* gene for the intracellular growth and pathogenicity of *T. gondii* through regulating mitochondrial function and the proline metabolism and provided a novel insight to reveal intracellular survival strategies of *T. gondii*.

**Graphical Abstract:**

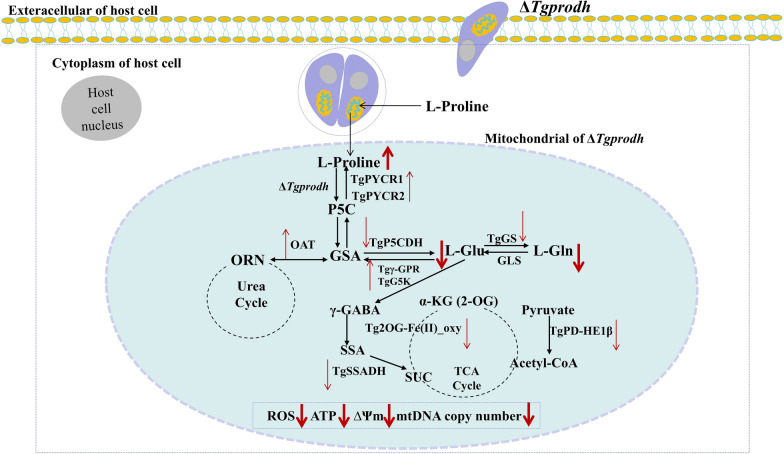

**Supplementary Information:**

The online version contains supplementary material available at 10.1186/s13071-025-06966-x.

## Background

*Toxoplasma gondii*, the causative agent of zoonotic toxoplasmosis, is an obligate intracellular parasite capable of infecting virtually all nucleated cells in diverse organisms [1]. The tachyzoites of *T. gondii* rapidly replicate via binary fission within the cells of intermediate or definitive hosts, triggering acute infections [2]. Upon immune pressure, tachyzoites transform into bradyzoites that persist encysted predominantly in muscles, lungs, and livers [3]. In the event that the host is under immunosuppression or has a weakened immune function, bradyzoites in tissue cysts are released and converted into rapidly multiplying tachyzoites, resulting in serious toxoplasmic encephalitis, myocarditis, and pneumonia [4]. In addition, primary infection with *T. gondii* during pregnancy can lead to infection of the unborn fetus via the placenta and is associated with miscarriage, stillbirth, or neurological deficits in the newborn [5].

As an auxotrophic parasite, *T. gondii* requires numerous amino acids and amino compounds that must be obtained from hosts during intracellular lytic cycle, including arginine, leucine, isoleucine, valine, methionine, threonine, and histidine [6–8]. Proline, a nonessential amino acid, can be catabolized to Δ1-pyrroline-5-carboxylate (P5C) by the proline dehydrogenase (PRODH), and P5C is further oxidized to glutamate by the P5C dehydrogenase (P5CDH). Glutamate then enters the tricarboxylic acid (TCA) cycle as a precursor of α-ketoglutarate (α-KG), or is converted into glutamine by glutamine synthetase (GS) [9]. In addition, glutamic-γ-semialdehyde (GSA), an isomer of P5C, can be catalyzed to ornithine by ornithine aminotransferase (OAT), and ornithine enters the urea cycle as a starting substance [10]. Recent studies have identified several enzymes and metabolites involved in the proline metabolism in bacteria (e.g., *Escherichia coli*, *Salmonella*, and *Pseudomonas*) [11–14], viruses (e.g., hepatitis C virus and yellow mosaic virus) [15–17] and parasites (e.g., *Trypanosoma brucei*, *Caenorhabditis elegans*) [18, 19], and provide the evidence of significance of the proline metabolism impacting the pathogen’s ability to survive and defense function. For example, *E. coli* can adjust the expression of proline utilization A (PutA) to enhance their ability to resist oxidative stress within the host [20], and the proline metabolism has been shown to be a crucial pathway by which *E. coli*, *P. putida*, *Bradyrhizobium japonicum*, and *Helicobacter pylori* acquire carbon and nitrogen sources under various auxotrophic conditions [21–24]. Hepatitis C virus decreases the concentration of proline through upregulating the proline oxidase to create a more favorable environment for replication and spread of the virus [16]. *T. brucei* utilizes the proline metabolism to supply energy for the oxidative phosphorylation process in the mitochondrion during its insect stages [18]. These studies indicated that different pathogens utilize the proline metabolism in different ways to obtain a survival advantage during the infection process. Yet, the proline metabolism in *T. gondii* is poorly understood. In the present study, the proline dehydrogenase (TGGT1_202340, named as *Tgprodh*) in *T. gondii* was identified by homology alignment of amino acid sequences, and the effect and mechanisms of the *Tgprodh* gene to the growth and pathogenicity of *T. gondii* were investigated.

## Methods

### Cells and parasites

The Vero cells (from the African green monkey kidney) were kindly provided by Prof. Xuefeng Qi from Northwest A&F University. Vero cells were grown in T-25 cell culture flasks containing Dulbecco’s modified Eagle’s medium (DMEM) supplemented with 10% fetal bovine serum (FBS) and 100 U/mL penicillin–streptomycin at 37 °C and 5% CO_2_ atmosphere.

The tachyzoites of *T. gondii* type I wild strain (RH) were gifted from Prof. Bang Shen in Huazhong Agricultural University. The parental RH strain (RH wild strain) was used as the vector to construct the knockout strain. All parasites were passaged in Vero cells. The infected cells were disrupted with a cell scraper (NEST Biotechnology, Wuxi, China), subjected to repeated aspiration with a 5-gauge needle (Shengguang Medical Products, Shanghai, China), and filtered by using a 5-µm pore size filter (Shanghai Xingya Purification Materials Factory, Shanghai, China). The number of purified tachyzoites was counted by using a cell-counting chamber under a microscope (KEYENCE, ChongQing, China).

### Construction and identification of the gene-edited strains

The endogenous labeling strain of the *Tgprodh* gene (*Tgprodh*-HA) was constructed by using the plasmids p10×hemagglutinin-tagged dihydrofolate reductase (HA-DHFR) plasmid (generously provided by Prof. Ningbo Xia from South China Agricultural University) and pSAG1::CAS9-U6::sgUPRT (Addgene no. 54,467, Miaoling Biology, Wuhan, China). Using the p10×HA-DHFR plasmid as a template to label 10× HA at the C-terminus of the *Tgprodh* gene, a pair of primers (*Tgprodh*-5′ untranslated region (UTR) and *Tgprodh*-3′UTR, Additional file 1: Table S1) containing 50-base pair (bp) sequences of the 5′ flank and 3′ flank sites in the *Tgprodh* gene were designed to amplify the homologous recombination fragment including the dihydrofolate reductase (DHFR) locus and the 10× hemagglutinin (HA) locus. A single guide RNA (sgRNA1) (Additional file 1: Table S1) targeting the site near the stop codon of the *Tgprodh* gene was used to replace the original guide RNA targeting the uracil phosphoribosyltransferase (*Tguprt*) gene in the pSAG1::CAS9-U6::sgUPRT plasmid to obtain the pSAG1::CAS9-U6::sgPRODH-1 plasmid by using a ClonExpress MultiS one-step cloning kit (Vazyme, Nanjing, China). Then, the pSAG1::CAS9-U6::sgPRODH-1 plasmid (5 μg) and the homologous recombination fragment (25 μg) were cotransfected into tachyzoites of the RHΔ*ku80* strain by using Gene Pulser Xcell™ electroporation system (Bio-Rad, CA, USA). The *Tgprodh*-HA strain was obtained by screening with 3 μmol/L pyrimethamine pressure.

To generate the knockout strain of the *Tgprodh* gene (RHΔ*prodh*), the full-length sequence of the *Tgprodh* gene (TGGT1_202340) was downloaded from the ToxoDB website (https://toxodb.org/), and a single guide RNA (sgRNA2) (Additional file 1: Table S1) targeting the coding region of the *Tgprodh* gene was used to replace the original guide RNA targeting the *Tguprt* gene in the pSAG1::CAS9-U6::sgUPRT plasmid to obtain the pSAG1::CAS9-U6::sgPRODH-2 plasmid. Two pairs of primers (*Tgprodh* 5′ knockout (KO) forward/reverse and *Tgprodh* 3′KO forward/reverse, Additional file 1: Table S1) were designed to amplify 1,000-bp sequences of the 5′ flank and 3′ flank sites in the *Tgprodh* gene of the parental RH strain, and the amplification fragments were inserted into the 5′ flank and 3′ flank sites in the DHFR resistance fragment of the pUPRT::DHFR-D plasmid (Addgene no. 58,528, Miaoling Biology, Wuhan, China), respectively. The plasmids pSAG1::CAS9-U6::sgPRODH-2 and pPRODH::DHFR-D (1:1, 30 μg) were cotransferred into the tachyzoite of the parental RH strain by using Gene Pulser Xcell™ electroporation system (Bio-Rad, CA, USA). The RHΔ*prodh* strain was obtained by screening with 3 μmol/L pyrimethamine pressure.

To generate the complementary strain (RHiΔ*prodh*) of the RHΔ*prodh* strain, the coding sequence of the *Tgprodh* gene was downloaded from the ToxoDB website (https://toxodb.org/), and a pair of primers (*Tgprodh*-CDS, Additional file 1: Table S1) were designed to amplify 1,458-bp sequences in the coding region of the *Tgprodh* gene from the parental RH strain. The complementary plasmid pUPRT::PRODH-D was constructed by replacing the DHFR resistance fragment with the coding region of the *Tgprodh* gene in the pUPRT::DHFR-D plasmid via a ClonExpress MultiS one-step cloning kit (Vazyme Biotech, Nanjing, China). The pSAG1::CAS9-U6::sgUPRT plasmid and the pUPRT::PRODH-D plasmid were cotransferred into the tachyzoites of the RHΔ*prodh* strain by using Gene Pulser Xcell™ electroporation system (Bio-Rad, CA, USA). The RHiΔ*prodh* strain was obtained by screening with 10 μmol/L 5′-fluorouracil and 3 μmol/L pyrimethamine pressure.

Identification of the *Tgprodh*-HA, RHΔ*prodh*, and RHiΔ*prodh* strains was performed by using polymerase chain reaction (PCR) according to previous studies [25]. Briefly, the genomic DNA (gDNA) of each strain was extracted by using a resin-based genomic DNA extraction kit (Sbsgenetech, Shanghai, China). Two (*Tgprodh* 5′UTR-HA and *Tgprodh* 3′UTR-HA), three (*Tgprodh*-gRNA*, Tgprodh*, and DHFR), and three (*Tgprodh*-identify, *Tguprt*, and DHFR) pairs of specific primers targeting the coding region of the *Tgprodh* gene (Additional file 1: Table S1) were designed to confirm the *Tgprodh*-HA, RHΔ*prodh*, and RHiΔ*prodh* strains, respectively. The total volume of the reaction system was 25.0 μL, containing 12.5 μL 2× Rapid Taq Master Mix, 0.4 μM of each primer, and 17.6 ng of gDNA. The amplification program consisted of initial predenaturation at 95 °C for 4 min, followed by 30 cycles of denaturation at 95 °C for 30 s, annealing at 58 °C for 30 s, extension at 72 °C for 2 min, and a final extension at 72 °C for 10 min.

### Western blot assay

In total, 1 × 10^7^ tachyzoites were lysed with 200 μL radioimmunoprecipitation assay (RIPA) lysis buffer (Beyotime, Shanghai, China). Then, the samples were analyzed by sodium dodecyl sulfate–polyacrylamide gel electrophoresis (SDS–PAGE) and subsequently transferred to the polyvinylidene fluoride (PVDF) membrane (Merck, New Jersey, USA). The membrane was blocked with 5% skimmed milk in Tris-buffered saline with Tween 20 (TBST) buffer at room temperature for 2 h, followed by incubation with mouse anti-HA monoclonal antibody (Sanying Biotechnology Co., Ltd., Wuhan, China) (1:1,000) and horseradish peroxidase (HRP)-conjugated goat anti-mouse immunoglobulin (Ig)G secondary antibody (Beyotime, Shanghai, China) (1:2,000). The TgPRODH protein band was visualized by using Super ECL Plus (Applygen, Beijing, China) and captured with a gel imaging system (SinSage Technology Co.,Ltd, Beijing, China) for analysis.

### Immunofluorescence assay

In total, 5 × 10^4^ Vero cells were seeded into 24-well plates for 24 h and infected with 1 × 10^3^ purified tachyzoites of the RH, RHΔ*prodh*, and RHiΔ*prodh* strains, respectively. At 6 h postinfection (hpi), the Vero cells were washed with PBS to remove uninvaded parasites and continually cultured for 24 h. Then, the cells were fixed with 4% paraformaldehyde (Biosharp, Beijing, China) for 20 min; permeabilized with 0.5% Triton X-100 (Solarbio, Beijing, China) for 15 min; incubated with mouse anti-HA (Sanying Biotechnology Co., Ltd., Wuhan, China), rabbit anti-TgCDPK1 (generously provided by Prof. Wang Quan from Shanghai Veterinary Research Institute), rabbit anti-NcPRODH (the antibodies were prepared by our laboratory), and MitoTraker for 1 h at 37 °C; and stained with the Alexa Fluor 594-conjugated goat anti-mouse IgG or Alexa Fluor 488-conjugated goat anti-rabbit IgG (Abcam, Cambridge, UK) for 1 h at 37 °C. The nuclei of cells and tachyzoites were stained with 4′,6-diamidino-2-phenylindole (DAPI) (Solarbio, Beijing, China) for 15 min at room temperature. The fields were recorded for each strain by using fluorescence microscopy (Leica DM4 B, Weztlar, Germany).

### Replication assay

In total, 2 × 10^5^ Vero cells were seeded into six-well plates for 24 h and infected with 1 × 10^5^ purified tachyzoites of the RH, RHΔ*prodh*, and RHiΔ*prodh* strains, respectively. At 6 h postinfection (hpi), the cells were washed with PBS to remove uninvaded parasites and continually cultured for 24 h and 48 h, respectively. The replication of parasites in Vero cells was determined according to previous studies [26, 27]. A total of 300 vacuoles of each strain were counted in the three independent experiments, and the number of tachyzoites in each vacuole was recorded. The replication rate was expressed as the percentage of vacuoles containing different numbers of tachyzoites and the average number of tachyzoites.

### Plaque assay

In total, 2 × 10^5^ Vero cells were seeded into six-well plates for 24 h and infected with 200 purified tachyzoites of the RH, RHΔ*prodh*, and RHiΔ*prodh* strains, respectively. At 7 days postinfection (dpi), the cells were washed with PBS to remove egressed parasites. Then, the cells were fixed with 4% paraformaldehyde (Biosharp, Beijing, China) at 4 °C for 20 min, stained with crystal violet for 15 min, gently washed three times with PBS, and dried at ambient temperature. The plaque size of parasites in Vero cells was measured according to previous studies [28, 29]. A total of three wells were infected with each strain, and the plaques of each well were recorded using an inverted microscope (KEYENCE, ChongQing, China). The plaque size was analyzed by using the Image J software (National Institutes of Health, Bethesda, USA).

### Invasion assay

In total, 5 × 10^4^ vero cells were seeded into 24-well plates for 24 h and infected with 1 × 10^3^ purified tachyzoites of the RH, RHΔ*prodh*, and RHiΔ*prodh* strains, respectively. At 6 hpi, the Vero cells were washed with PBS to remove uninvaded parasites and continually cultured for 24 h. Then, the cells were fixed with 4% paraformaldehyde (Biosharp, Beijing, China) for 20 min, permeabilized with 0.5% Triton X-100 (Solarbio, Beijing, China) for 15 min, incubated with rabbit anti-TgSAG1 (the antibodies were prepared by our laboratory) for 1 h at 37 °C, and, finally, stained with the Alexa Fluor 488-conjugated goat anti-rabbit IgG (Abcam, Cambridge, UK) for 1 h at 37 °C. The nuclei of cells and tachyzoites were stained with DAPI (Solarbio, Beijing, China) for 15 min at room temperature. In total, three wells were infected with each strain. The invasion rate of parasites in Vero cells was calculated as described previously [30]. At least three fields were recorded for each strain by using fluorescence microscopy (Leica DM4 B, Weztlar, Germany), and the invasion rate was represented as the percentage of infected host cells in the total number of host cells in each field of view.

### Egress assay

In total, 5 × 10^4^ Vero cells were seeded into 24-well plates for 24 h and infected with 1 × 10^3^ purified tachyzoites of the RH, RHΔ*prodh*, and RHiΔ*prodh* strains, respectively. At 6 hpi, the Vero cells were washed with PBS to remove uninvaded parasites and continually cultured for 48 h. After the extracellular tachyzoites were removed with PBS, and the intracellular tachyzoites were stimulated with calcium ionophore A23187 (3 μmol/mL) (Aladdin, Shanghai, China) for 2 min. Then, the cells were fixed with 4% paraformaldehyde (Biosharp, Beijing, China) for 20 min and permeabilized with 0.5% Triton X-100 (Solarbio, Beijing, China) for 15 min. Intracellular and extracellular tachyzoites were incubated with rabbit anti-TgSAG1 (the antibodies were prepared by our laboratory) and Alexa Fluor 488-conjugated goat anti-rabbit IgG (Abcam, Cambridge, UK) for 1 h at 37 °C. The nuclei of cells and tachyzoites were stained with DAPI (Solarbio, Beijing, China) for 15 min at room temperature. Three wells were infected with each strain. The egress rate of each stain was calculated as described previously [30]. In each of three independent experiments, at least 100 parasitophorous vacuoles (PV) of each strain were observed by using fluorescence microscopy (Leica DM4 B, Weztlar, Germany), and the egress rate was represented as the percentage of PV with egress tachyzoites in the total number of 100 PV.

### Detection of proline metabolites

In total, 2 × 10^6^ Vero cells were seeded into a T25 cell culture flask for 24 h and infected with 1 × 10^6^ purified tachyzoites of the RH, RHΔ*prodh*, and RHiΔ*prodh* strains, respectively. At 48 hpi, cells were collected to purify the tachyzoites as above. The contents of l-proline (L-Pro), l-glutamic acid (L-Glu), and glutamine (Gln) were determined by using proline (Pro) content assay kit (Solarbio, Beijing, China), glutamic acid (Glu) content kit-visible color, and glutamine (Gln) content kit (Grace Biotechnology, Suzhou, China) according to the instructions. Three biological replicates of each strain were detected.

### Transmission electron microscopy (TEM) analysis

TEM analysis was performed as described previously [31]. Briefly, 2 × 10^6^ Vero cells were seeded into T25 cell culture flask for 24 h and then infected with 1 × 10^6^ purified tachyzoites of the RH, RHΔ*prodh*, and RHiΔ*prodh* strains, respectively. At 6 hpi, the cells were washed with PBS to remove uninvaded parasites and continually cultured for 18 h, respectively. Then, the cells were digested with 0.25% trypsin (Mishushengwu, Xian, China) for 1 min at 37 °C, and centrifuged at 4 ℃ for 10 min. The pellets were fixed with electron microscope fixative at 4 °C for 30 min and embedded in resin. Ultrathin sections with a thickness of 60–80 nm were obtained from the resin blocks by using a Leica EM UC7 ultramicrotome (Leica, Wetzlar, Germany) and scanned under a HITACHI HT7700 80 kv transmission electron microscope (Hitachi, Tokyo, Japan). TEM analysis was performed by Wuhan Saiweier Biotechnology Co., Ltd. In total, three biological replicates of each strain were analyzed.

### Determination of ROS levels

In total, 2 × 10^6^ Vero cells were seeded into T25 cell culture flask for 24 h and infected with 1 × 10^6^ purified tachyzoites of the RH, RHΔ*prodh*, and RHiΔ*prodh* strains, respectively. At 48 hpi, cells were collected to purify the tachyzoites as above. The ROS levels in tachyzoites were detected by using the reactive oxygen species (ROS) assay kit (Beyotime, Shanghai, China). Briefly, the tachyzoites were labeled with 2′,7′-dichlorofluorescein diacetate (DCFH-DA) for 20 min at 37 °C. Then, the tachyzoites were washed three times with serum-free DMEM and resuspended in 200 μL of serum-free DMEM. The intracellular fluorescence intensity was recorded by using a multifunctional microplate reader (excitation/emission wavelengths of 480/525 nm) (Spark, Shanghai, China). In total, three biological replicates of each strain were detected. The ROS levels in the RHΔ*prodh* and RHiΔ*prodh* strains were expressed as the fluorescence multiples relative to the parental RH strain.

### Measurement of the ATP content

A total of 2 × 10^6^ Vero cells were seeded into T25 cell culture flask for 24 h and infected with 1 × 10^6^ purified tachyzoites of the RH, RHΔ*prodh*, and RHiΔ*prodh* strains, respectively. At 48 hpi, cells were collected to purify the tachyzoites as above. The ATP content in tachyzoites was measured by using an ATP assay kit (Beyotime, Shanghai, China). Briefly, the tachyzoites of each strain were lysed by 200 μL of ATP lysis solution for 10 min at 4 ℃, and then, the supernatant of the sample was obtained by centrifugation at 4 ℃. The ATP content in the supernatant was recorded by a multifunctional microplate reader (Spark, Shanghai, China); three biological replicates of each strain were measured. The ATP standard solution was diluted with the ATP detection lysis solution to concentrations of 0.01, 0.03, 0.1, 0.3, 1, 3, and 10 μM, respectively, and the standard curve of the ATP content was plotted on the basis of the luminescence corresponding to these concentrations. The ATP concentrations in the RH, RHΔ*prodh*, and RHiΔ*prodh* strains were calculated on the basis of the standard curve.

### Analysis of mitochondrial membrane potential (∆Ψm)

A total of 2 × 10^6^ Vero cells were seeded into T25 cell culture flask for 24 h and infected with 1 × 10^6^ purified tachyzoites of the RH, RHΔ*prodh*, and RHiΔ*prodh* strains, respectively. At 48 hpi, cells were collected to purify the tachyzoites as above. The mitochondrial membrane potential of tachyzoites was detected according to the instructions of the Mito-Tracker Red CMXRos kit (Beyotime, Shanghai, China). Briefly, the tachyzoites of each strain were coincubated with Mito-Tracker Red CMXRos (100 nM) at 37 ℃ for 30 min and then centrifuged at room temperature for 10 min. The pellets were resuspended in 200 μL of fresh cell culture medium prewarmed at 37 ℃; three biological replicates of each strain were detected. The fluorescence intensity of each sample was recorded by a multifunctional microplate reader (excitation/emission wavelengths of 579/599 nm) (Spark, Shanghai, China). The mitochondrial membrane potential of RHΔ*prodh* and RHiΔ*prodh* strains was presented as the fold change in fluorescence relative to the parental RH strain.

### Detection of mitochondrial DNA (mtDNA) copy number

A total of 2 × 10^6^ Vero cells were seeded into T25 cell culture flask for 24 h and infected with 1 × 10^6^ purified tachyzoites of the RH, RHΔ*prodh*, and RHiΔ*prodh* strains, respectively. At 48 hpi, cells were collected to purify the tachyzoites as above. The genomic DNA of tachyzoites was extracted by using a resin-based genomic DNA extraction kit (Sbsgenetech, Shanghai, China) according to the manufacturer’s instructions. The primers (Additional file 1: Table S1) for the *T. gondii* 18S ribosomal (*Tg18SrRNA*) gene and cytochrome oxidase 1 (*Tgcox1*) used in the quantitative polymerase chain reaction (qPCR) were designed by using DNAMAN 7.0 (Lynnon biosoft, Quebec, Canada). The total volume of the reaction system was 20.0 μL, containing 10 μL 2× Universal SYBR Green Fast RT-qPCR Mix (ABclonal, Wuhan, China), 0.25 μM forward primer, 0.25 μM reverse primer, and 25.0 ng complementary DNA (cDNA). The amplification program consisted of initial predenaturation at 95 °C for 3 min, followed by 45 cycles of denaturation at 95 °C for 10 s, annealing at 56 °C for 30 s, and extension at 72 °C for 1 min. The *Tg18SrRNA* gene was used as a reference gene to normalize the expression level of mitochondrial DNA copy numbers (*Tgcox1*) in *T. gondii*. In total, three biological replicates and three technical replicates were set up for each strain.

### Reverse transcriptase quantitative polymerase chain reaction (RT-qPCR)

In total, 2 × 10^6^ Vero cells were seeded into T25 cell culture flask for 24 h and infected with 1 × 10^6^ purified tachyzoites of the RH, RHΔ*prodh*, and RHiΔ*prodh* strains, respectively. At 48 hpi, cells were collected to purify the tachyzoites as above. The total RNA was extracted from purified tachyzoites using TRIzol reagent (Invitrogen, MA, USA), and cDNAs were obtained by using a reverse transcription kit (Accurate Biotechnology Co., Ltd, Hunan, China). The gene primers (Additional file 1: Table S1) of enzymes regulating the proline metabolism in *T. gondii*, namely, pyrroline-5-carboxylate reductase 1 (*Tgpycr1*), pyrroline-5-carboxylate reductase 1 (*Tgpycr2*), ornithine aminotransferase (*Tgoat*), glutamate 5-kinase (*Tgg5k*), gamma-glutamyl phosphate reductase (*Tgγ-gpr*), pyrroline-5-carboxylate dehydrogenase (*Tgp5cdh*), glutamine synthetase (*Tggs*), succinate-semialdehyde dehydrogenase (*Tgssadh*), pyruvate dehydrogenase complex subunit pd-he1beta (*Tgpd-he1β*), 2OG-Fe(II) oxygenase (*Tg2OG-Fe(II)_oxy*), and genes related to the integrity of mitochondria in *T. gondii*, namely, autophagy-related protein 3 (*Tgatg3*), dynamin-related protein (*Tgdrpc*), and ribosomal protein (*Tgms*35), for RT-qPCR were designed by using DNAMAN7.0 (Lynnon biosoft, Quebec, Canada). The total volume of the reaction system for RT-qPCR was 20.0 μL, containing 10 μL of 2× Universal SYBR Green Fast RT-qPCR Mix (ABclonal, Wuhan, China), 0.25 μM of forward primer, 0.25 μM of reverse primer, and 12.5 ng of cDNA. The amplification program consisted of initial predenaturation at 95 °C for 3 min, followed by 45 cycles of denaturation at 95 °C for 10 s, annealing at 50–60 °C for 30 s, and extension at 72 °C for 1 min. The *β-tubulin* was used as a reference gene to normalize the expression levels of the genes; three biological replicates and three technical replicates were conducted for each strain.

### Animal assay

The protocol of the animal assay was reviewed and approved by the Institutional Animal Care and Use Committee of Northwest A&F University. Specific-pathogen-free (SPF) female Kunming mice (7 weeks old, weighing 35 ± 0.44 g) were purchased from Chongqing Tengxin Biotechnology Co., Ltd. All animals were maintained in an environment of a temperature of 22 °C, a relative humidity ranging from 40 to 70%, a 12-h light/12-h dark cycle, and free access to food and water.

A total of 40 mice were selected for assessing virulence of each of three *T. gondii* strains (RH, RHΔ*prodh* and RHiΔ*prodh*) and randomly divided into four groups: the PBS control group (10 mice), the RH-infected group (10 mice), the RHΔ*prodh*-infected group (10 mice), and the RHiΔ*prodh*-infected group (10 mice). A total of 1,000 purified tachyzoites of each strain were injected intraperitoneally into ten mice, respectively, and the mice were feed for 10 dpi. The symptoms and the number of dead mice were recorded each day. The survival rate of the mice was presented as survival curves using Prism 8 (GraphPad Software Inc.,La Jolla, CA, USA).

A total of 20 mice were selected for determining the tissue load of each of three *T. gondii* strains in mice, and randomly divided into four groups: the PBS control group (five mice), the RH-infected group (five mice), the RHΔ*prodh*-infected group (five mice), and the RHiΔ*prodh*-infected group (five mice). A total of 1 × 10^3^ purified tachyzoites of each strain were injected intraperitoneally into five mice, respectively. The symptoms and the number of dead mice were recorded each day. At 7 dpi, all live mice were euthanized with pentobarbital sodium, and the blood, peritoneal fluid, heart, liver, spleen, lung, and kidney of each mouse were collected. The gDNA were extracted by using TIANamp Genomic DNA Kit (TIANGENE, Beijing, China). The tissue load of parasites was detected by using qPCR. The total volume of the reaction system was 20.0 μL, containing 10 μL of 2× Universal SYBR Green Fast RT-qPCR Mix (ABclonal, Wuhan, China), 0.25 μM of forward primer, 0.25 μM of reverse primer, and 25.0 ng of cDNA. The amplification program consisted of initial predenaturation at 95 °C for 3 min, followed by 45 cycles of denaturation at 95 °C for 10 s, annealing at 55–60 °C for 30 s, and extension at 72 °C for 1 min. The tissue load of parasites was presented as the relative expression normalized to the *T. gondii* *B1* gene (Additional file 1: Table S1) [32].

### Statistical analysis

All data were analyzed by using GraphPad Prism 8.0.2 (San Diego, CA, USA). The Student’s *t*-test was used to evaluate the intergroup differences. The results were expressed as means ± SD. *P* < *0.05* was considered to be a significant difference.

## Results

### TgPRODH protein is expressed in *T. gondii*

**Fig. 1:**
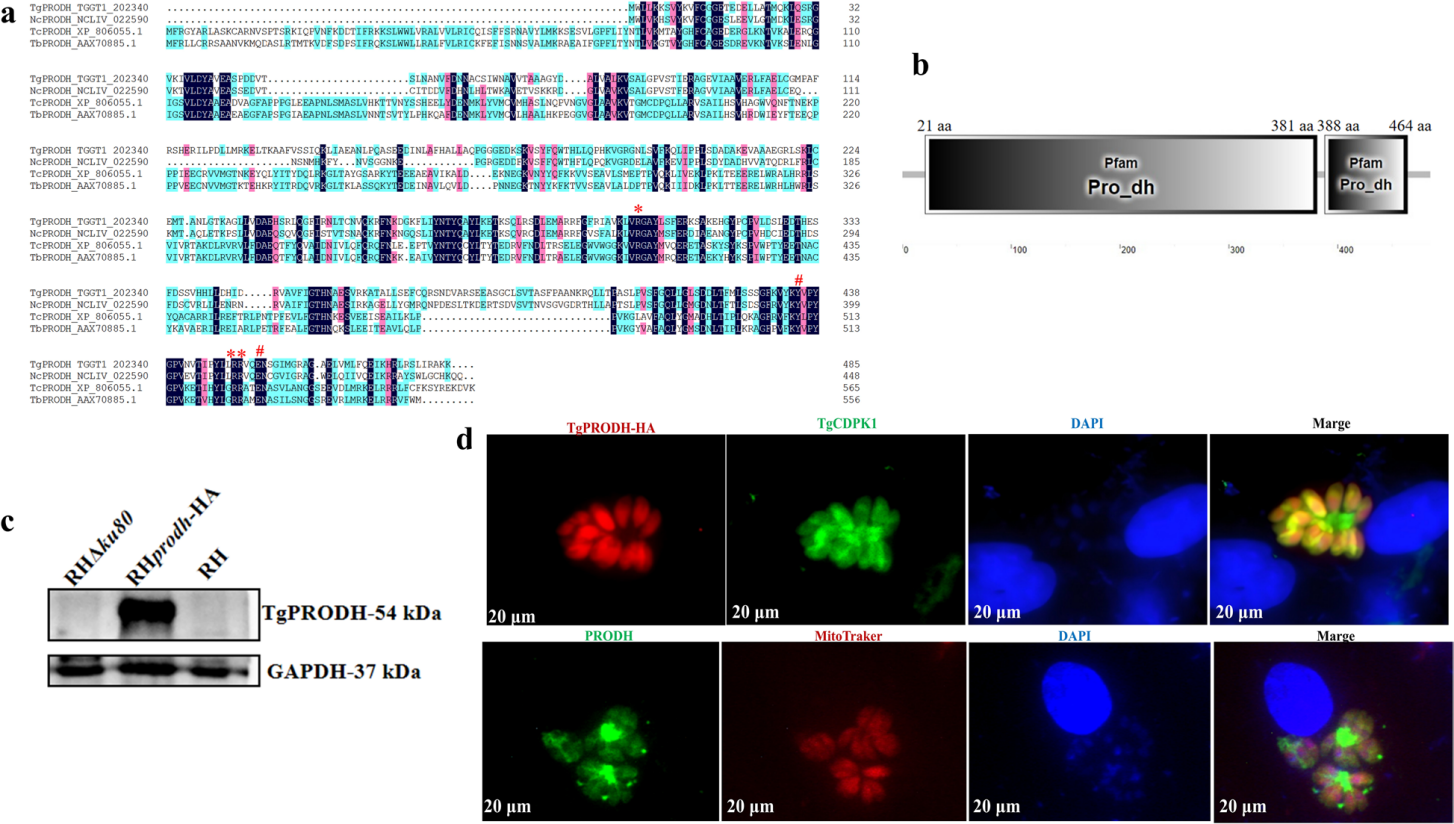
Sequence alignment, structure, and subcellular localization of TgPRODH were shown in *T. gondii*. **a**, Sequence alignment of TgPRODH, NcPRODH, TcPRODH, and TbPRODH. Three arginine (*) and two glutamic acid (**#**) sites were identified in TgPRODH. **b**, The proline oxidase active domains of TgPRODH predicted by using the SMART database (http://smart.embl-heidelberg.de/). Two proline oxidase active sites, located at 21 aa–381 aa and 388 aa–464 aa, were identified. **c**, The protein level of TgPRODH was detected by using western blot. The endogenous labeling strain (*Tgprodh*-HA) was used as the experimental group, while the RH*ku80* and RH wild strains were used as the control groups. The PVDF membrane was sectioned into two segments. One section was incubated with HA mouse monoclonal antibody, and the other was incubated with GAPDH rabbit monoclonal antibody. **d**, Location of TgPRODH in *T. gondii* by using immunofluorescence with mouse anti-HA (red), rabbit anti-TgCDPK1 (green), rabbit anti-NcPRODH (green), and MitoTraker (red). The *Tgprodh*-HA strain was used as the experimental group, and the RH*ku80* strain was used as the control groups. The scale bar is 20 μm

Sequencing and alignment showed that the nucleotide sequence of the *Tgprodh* (TGGT1_202340) gene contained 1,458 base pairs (bp), encoding 485 amino acids (aa) with variations of five amino acids to TgRUB PRODH (TGRUB_202340). Notably, the amino acid sequence of the *Tgprodh* gene has the highest sequence identity with that of *N. caninum* Liverpool (NCLIV_022590, 63.71%) (Additional file 2: Fig. S1). Phylogenetic analysis revealed that the *Tgprodh* gene was more closely related to the proline dehydrogenase (*Ncprodh*) gene of *N. caninum* (Additional file 3: Fig. S2). A total of three arginine sites (Arg^303^, Arg^450^ and Arg^451^) and two glutamic acid sites (Glu^435^, Glu^454^) of TgPRODH were identified (Fig. 1a) by using the InterProScan database (http://www.ebi.ac.uk/Tools/pfa/iprscan/). Sites of Arg^450^ and Arg^451^ in TgPRODH were closely associated with substrate binding, while sites of Arg^303^, Glu^435^, and Glu^454^ played critical roles for flavin adenine dinucleotide (FAD) binding. Interestingly, there arginine sites (Arg^303^, Arg^450^ and Arg^451^) and two glutamic acid sites (Glu^435^, Glu^454^) of NcPRODH were consistent with those in TgPRODH. In total, two proline oxidase active domains of the *Tgprodh* gene, located at amino acid positions 21 aa–381 aa and 388 aa–464 aa, were predicted by using the SMART database (http://smart.embl-heidelberg.de/) (Fig. 1b). To characterize the subcellular localization of TgPRODH, a 10× HA tag was introduced at the C-terminus of the parental RH strain by using clustered regularly interspaced short palindromic repeats/CRISPR-associated protein 9 (CRISPR–Cas9) technology (Additional file 4: Fig. S3a, b). The expression of the proline dehydrogenase (TgPRODH) in *T. gondii* was confirmed by using Western blot with mouse anti-HA (Fig. 1c). Furthermore, the immunofluorescence assay with rabbit anti-TgCDPK1 and MitoTraker confirmed that TgPRODH localized in the cytoplasm and mitochondria (Fig. 1d).

### TgPRODH is indispensable for growth and virulence of tachyzoites

**Fig. 2:**
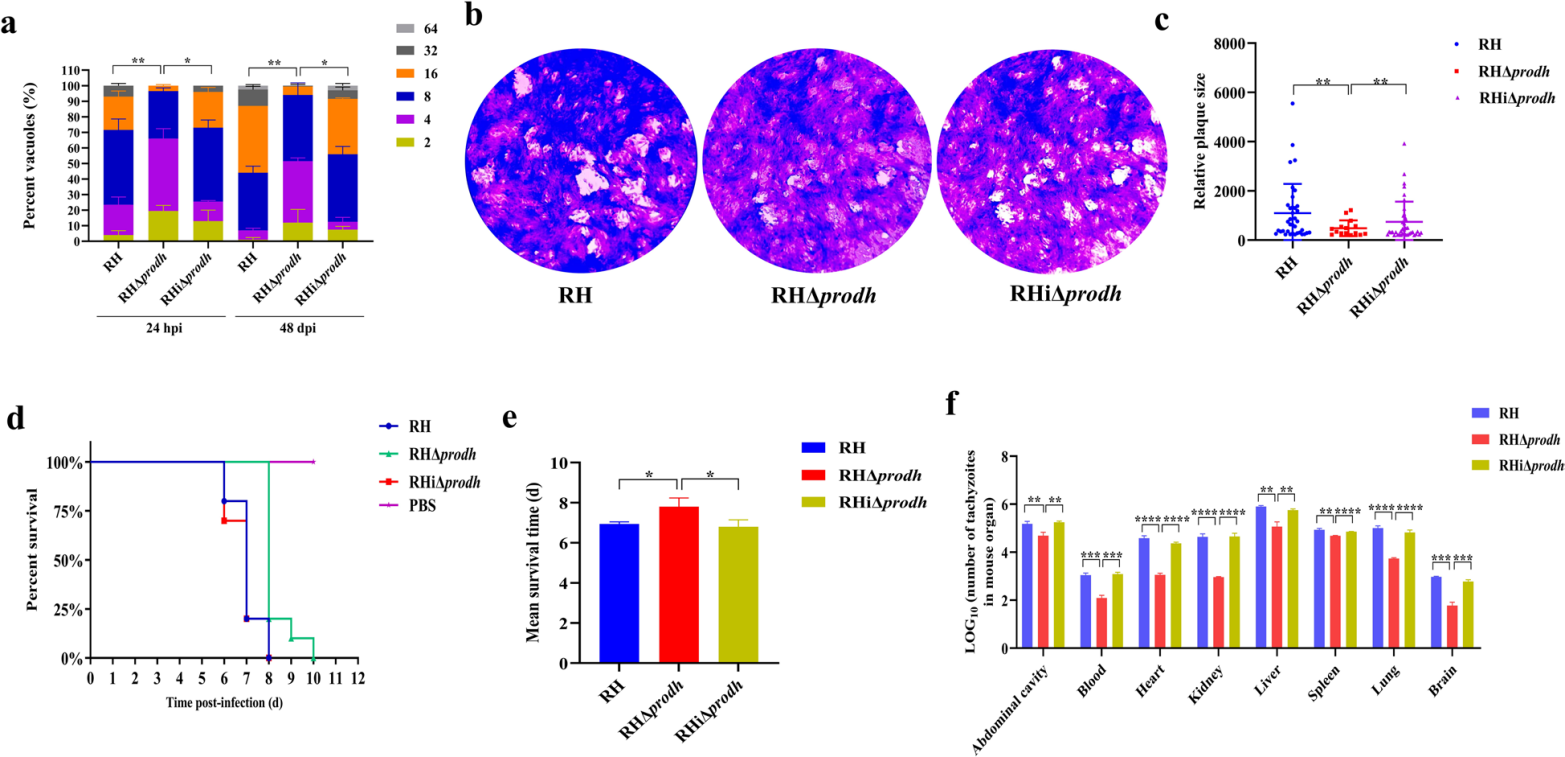
Deletion of *Tgprodh* gene resulted in the growth inhibition of *T. gondii *in vitro and reduced pathogenicity in vivo. **a**, The intracellular proliferation differences of the tachyzoites. **b**, The growth abilities of the tachyzoites were evaluated by using plaque assays. **c**, The sizes of the plaques for the tachyzoites were analyzed by Image J software. **d**, Comparison of the virulence of the tachyzoites to mice. **e**, The average survival time of mice infected with the tachyzoites. **f**, The tissue loads of mice infected with the tachyzoites. The differences were analyzed by Student’s *t*-test. *P < 0.05, **P < 0.01, ***P < 0.001, ****P < 0.0001

To investigate the function of TgPRODH, the *Tgprodh* gene was knocked out by using CRISPR–Cas9-mediated homologous recombination in the RH strain (Additional file 4: Fig. S3). PCR (Fig. S3c, d), western blot (Fig. S4a), and immunofluorescence (Fig. S4b) (Additional file 4: Fig. S3; Additional file 5: Fig. S4) confirmed that the gene deletion mutant (RHΔ*prodh*) was successfully constructed after pyrimethamine selection. In Vero cells, although the invasion efficiency (Additional file 6: Fig. S5a, b) and the egress rate from the PVs within 3 min (Additional file 6: Fig. S5c, d) were similar between RHΔ*prodh* and the parental RH strain, the number of tachyzoites in parasitophorous vacuole was significantly decreased at 24 hpi and 48 hpi (Fig. 2a), and the plaque formation areas by continuous 7-day culture were obviously reduced for the RHΔ*prodh* strain (Fig. 2b, c). In vivo study using the Kunming mouse model showed that the virulence of RHΔ*prodh* strain was significantly weakened (Fig. 2d, e), and the parasite loads of the RHΔ*prodh* strain were significantly decreased in the heart, liver, spleen, lung, kidney, and brain tissues compared with the parental RH strain (Fig. 2f). Notably, the complementary strain (RHiΔ*prodh*) (Additional file 4: Fig. S3e, f; Additional file 5: Fig. S4a, b) rescued the proliferative (Fig. 2a) and viability abilities (Fig. 2b, c) in Vero cells and virulence (Fig. 2d–f) in the mouse model. These results suggest that the *Tgprodh* gene is indispensable for the intracellular replication and virulence of *T. gondii* tachyzoite.

### TgPRODH is crucial for maintaining the structure and function of mitochondria in *T. gondii*

**Fig. 3:**
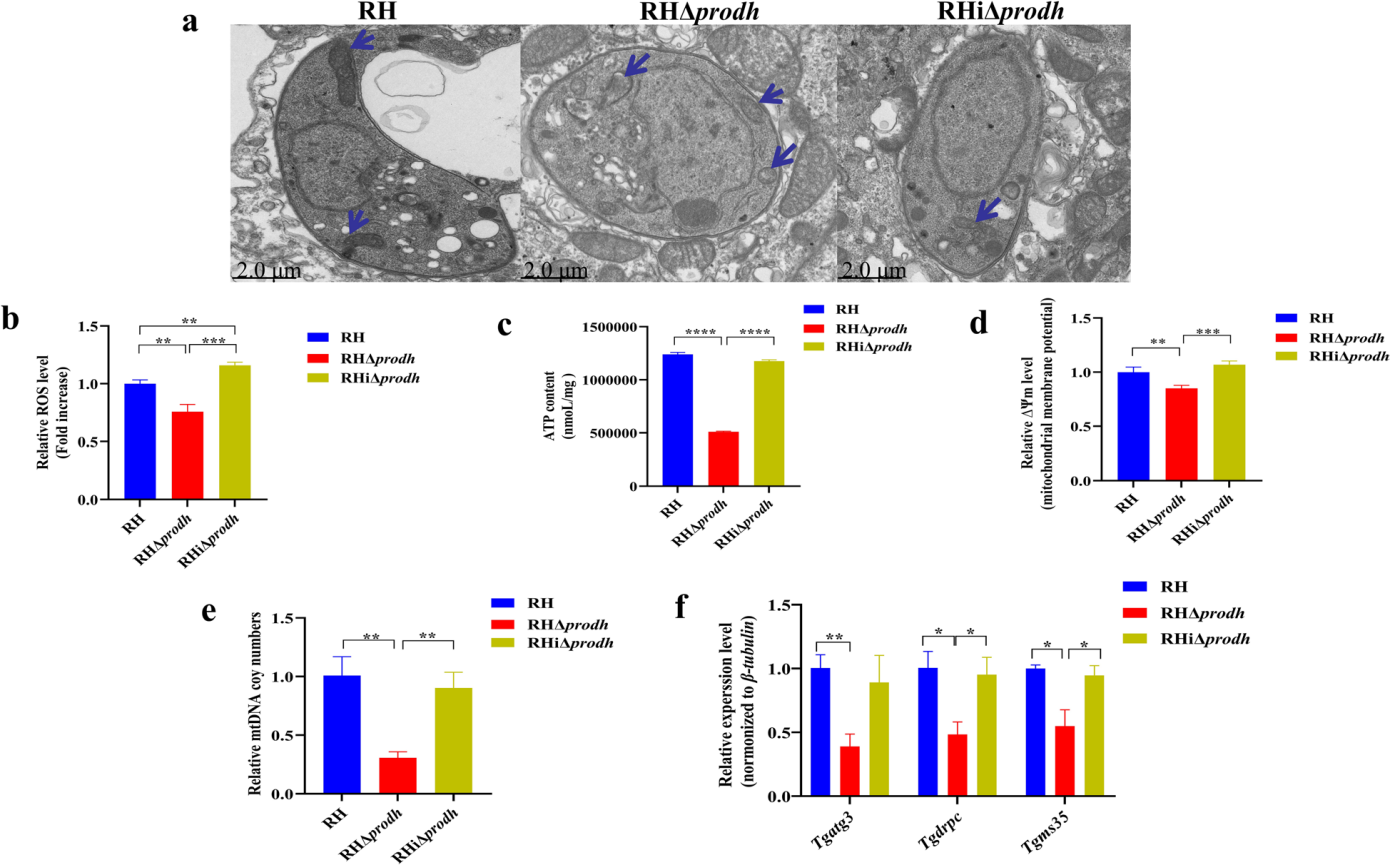
Deletion of *Tgprodh* gene resulted in mitochondrial damage in *T. gondii*. **a**, Electron microscopy analysis of tachyzoites in *T. gondii*. The mitochondria of tachyzoites in the RH strain showed a typical lasso shape (blue arrow). **b**, The ROS levels of tachyzoites. **c**, The ATP content of the tachyzoites. **d**, The mitochondrial membrane potential of tachyzoites. **e**, The mitochondrial DNA copy number of tachyzoites. **f**, The mRNA levels of genes related to mitochondrial integrity in the tachyzoites. Three independent experiments were conducted, and the differences were analyzed by Student’s *t*-test. *P < 0.05, **P < 0.01, ***P < 0.001, ****P < 0.0001

The proline dehydrogenase has been reported to be a mitochondrial inner-membrane protein [33], and deletion of the proline dehydrogenase led to mitochondrial defects in *C. elegans*, *T. cruzi*, and *A. thaliana* [34–36]. The mitochondria dysfunction has also been reported to result in disorder of the proline metabolism in *C. elegans* [37], and the biosynthesis of proline has been found to be impaired in fibroblasts owing to mitochondrial defects [38]. To determine whether the *Tgprodh* gene was associated with the mitochondrial function in *T. gondii*, the morphological feathers of the mitochondria were observed in the RHΔ*prodh* strain and the parental RH strain by using the electron microscope analysis. In the RH strain, the mitochondria showed a typical lasso-shaped or open lasso shape morphology, consistent with previous reports [31, 39–41]. By contrast, two different mitochondrial phenotypes, spherical or broken, are identified in the RHΔ*prodh* strain (Fig. 3a). It is worth noting that, in the RHΔ*prodh* strain, the mitochondria were significantly swollen and the inner ridge structure had disappeared or shrank into a spherical shape (Fig. 3a). Furthermore, compared with the parental RH strain, the levels of ROS, ∆Ψm, ATP content, and mtDNA copy numbers in the RHΔ*prodh* strain were significantly decreased (Fig. 3b–e), and the mRNA levels of three representative genes (*Tgatg3*, *Tgdrpc*, and *Tgms35*) responsible for maintaining mitochondrial integrity were also significantly decreased in this strain (Fig. 3f). However, the complementation of the *Tgprodh* gene restored the morphological and functional defects of mitochondria in the RHΔ*prodh* strain (Fig. 3a–f). These results suggest that the *Tgprodh* gene is responsible for the maintenance of the structure and function of the mitochondria in *T. gondii*.

### TgPRODH is essential for the proline metabolism

**Fig. 4:**
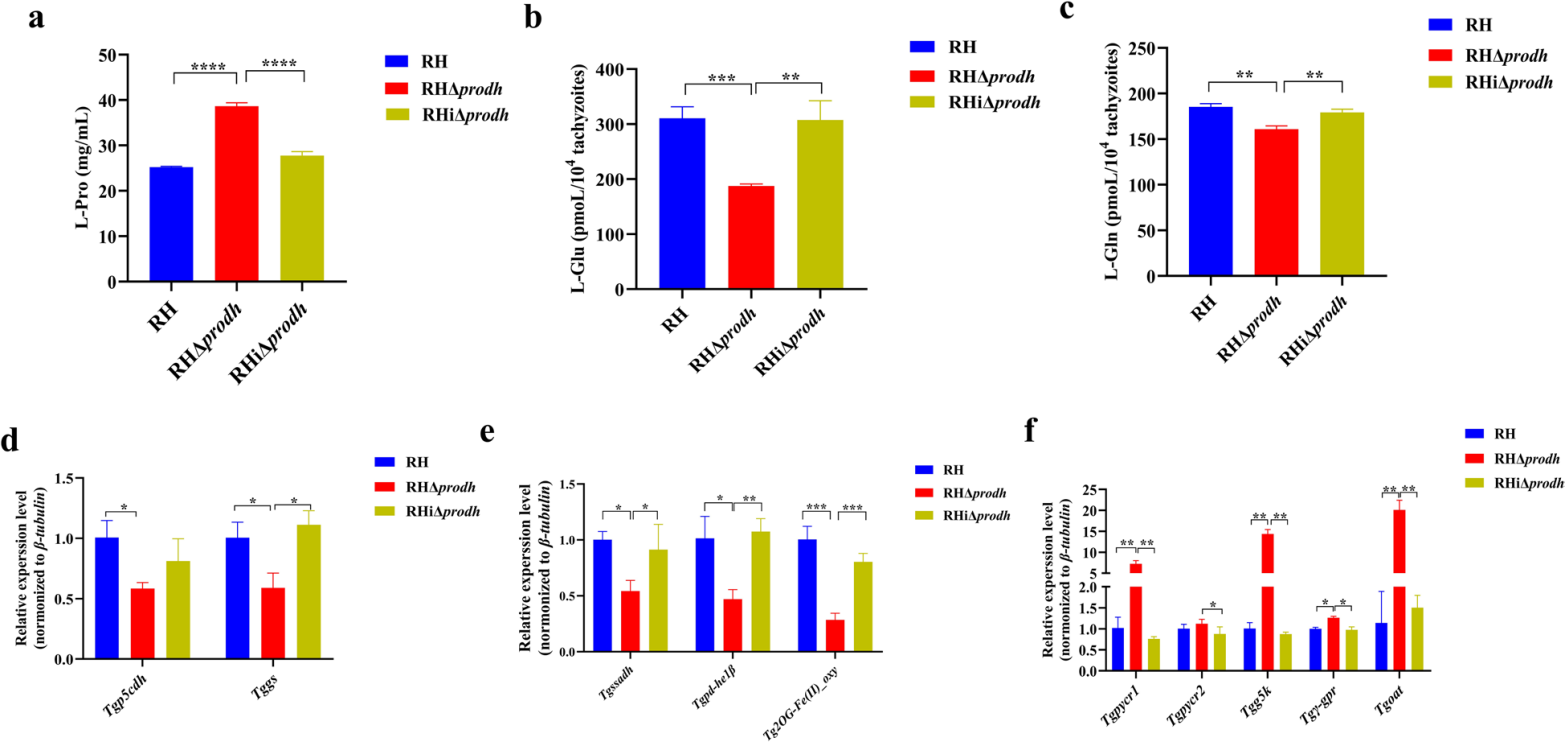
Deletion of *Tgprodh* gene resulted in disorder of proline metabolism in *T. gondii*. **a**, The concentration of l-proline in the tachyzoites. **b**, The concentration of l-glutamic acid in the tachyzoites. **c**, The concentration of l-glutamine in the tachyzoites. **d**, **e**, The mRNA levels of enzymes involved into the proline catabolism in the tachyzoites. **f**, The mRNA levels of enzymes involved into the proline anabolism in the tachyzoites. Three independent experiments were conducted, and the differences were analyzed by Student’s *t*-test. *P < 0.05, **P < 0.01, ***P < 0.001, and ****P < 0.0001

The proline dehydrogenase has been reported to regulate the proline metabolism in eukaryotes [42, 43]. We then asked whether the *Tgprodh* gene regulates the proline metabolism of *T. gondii*. To address this issue, the levels of L-Pro, L-Glu, and L-Gln were determined in the RHΔ*prodh* strain and the parental RH strain by using a microassay kit. Compared with the parental RH strain, the concentration of L-Pro was significantly increased in the RHΔ*prodh* strain (Fig. 4a), but the concentrations of L-Glu and L-Gln were significantly decreased in the RHΔ*prodh* strain (Fig. 4b, c). Protein interaction analysis using the STRING database (https://string-db.org/) showed ten genes involved in the proline metabolism were identified to interact with the *Tgprodh* gene in *T. gondii*, namely *Tgpycr1* (TGGT1_236070), *Tgpycr2* (TGGT1_271610), *Tgoat* (TGGT1_269110), *Tgg5k* (TGGT1_265010), *Tgγ-gpr* (TGGT1_270550), *Tgp5cdh* (TGGT1_288450), *Tggs* (TGGT1_273490), *Tgssadh* (TGGT1_257480), *Tgpd-he1β* (TGGT1_272290), and *Tg2OG-Fe(II)_oxy* (TGGT1_221270). RT-qPCR analysis confirmed that the mRNA levels of several genes (e.g., *Tgpycr1*, *Tgpycr2*, *Tgoat*, *Tgg5k*, and *Tgγ-gpr*) involved in the proline anabolism were significantly upregulated in the RHΔ*prodh* strain (Fig. 4f), while the opposite results were observed for the expression of several genes (e.g., *Tgp5cdh,* *Tggs*, *Tgssadh*, *Tgpd-he1β*, and *Tg2OG-Fe(II)_oxy*) involved in the proline catabolism (Fig. 4d, e). Conversely, the complementation of the *Tgprodh* gene recovered the concentrations of products in proline metabolism and expression of genes above in the RHΔ*prodh* strain (Fig. 4a–f). These results suggested that the *Tgprodh* gene plays a vital regulatory role in the proline metabolism of *T. gondii.*

## Discussion

**Fig. 5:**
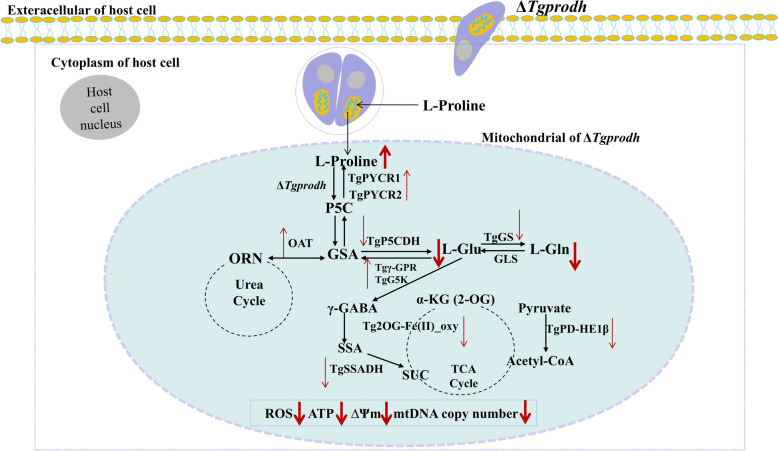
A hypothesized mechanism diagram showed impacts of the *Tgprodh* gene on the mitochondria and proline metabolism in *T. gondii*. On the one hand, disruption of the *Tgprodh* gene caused damage to the mitochondrial morphology, decreased membrane potential and mtDNA copy numbers, reduced the production of ATP and ROS, and downregulated the gene expression for maintaining mitochondrial integrity of *T. gondii*. On the other hand, disruption of the *Tgprodh* gene downregulated the mRNA levels of proline catabolic enzymes (*Tgp5cdh*, *Tggs*, *Tgssadh*, *Tgpd-he1β*, and *Tg2OG-Fe(II)_oxy*), led to accumulation of proline, and decreased the contents of L-Glu and L-Gln in *T. gondii*, and then, L-Glu was unable to enter the TCA cycle. Conversely, to obtain nutrients and energy from proline metabolism to maintain the intracellular growth of tachyzoites, the *Tgprodh*-deficient strain may have utilized the L-Glu, L-Gln, and ornithine in the cytoplasm of *T. gondii* to synthesize l-proline by upregulating the mRNA levels of proline anabolism enzymes (*Tgpycr1*, *Tgpycr2*, *Tgoat*, *Tgg5k*, and *Tgγ-gpr*). The *Tgprodh* gene is important for intracellular growth of tachyzoites by regulating the proline metabolism and mitochondrial function of the parasite

As an intracellular parasite, *T. gondii* regulates multiple metabolic pathways to meet its own requirements for energy and nutrients during its parasitism [44–46]. Over the past few decades, the proline metabolism has been identified as an important pathway for the energy source of intracellular pathogens, including protozoan parasites (*T. brucei*, *T. cruzi*) [18, 47]. The present study found that TgPRODH, the first rate-limiting enzyme in proline metabolism, was encoded by the *Tgprodh* gene in *T. gondii* and was identified to be indispensable for the tachyzoite growth of *T. gondii* through regulating proline metabolism and mitochondrial function of the parasite (Fig. 5).

In the present study, deletion of the *Tgprodh* gene in *T. gondii* resulted in significant growth inhibition (including proliferation and plaque formation) in vitro, and this phenomenon was also found for the *Tbprodh* gene in *T. brucei* [48]*.* Furthermore, the present study also observed that deletion of the *Tgprodh* gene in *T. gondii* resulted in weakened pathogenicity in mice and the alleviation of tissue damage simultaneously, consistent with the deletion of the PutA gene in *C. albicans* [49]. To confirm the function of the *Tgprodh* gene in *T. gondii*, the complementary strain of the *Tgprodh* gene knockout strain was constructed in the present study, and the complementation of the *Tgprodh* gene restored the defects in the biological phenotype. However, the egress rate and invasion rate of the *Tgprodh* gene knockout strain were similar to that of the parental RH strain. These results suggested that the *Tgprodh* gene is crucial for the pathogenicity of *T. gondii* with respect to the growth but not the egress and invasion of this parasite.

In the present study, the TgPRODH protein identified in *T. gondii* contained the conserved regions of the proline oxidase family and the FAD binding domain, consistent with those found in the proline dehydrogenase gene of several other pathogens (e.g., *T. cruzi*, *T. brucei*, and *E. coli*). The FAD domain has been reported to participate in the oxidation and reduction processes through accepting hydrogen atoms and transferring electrons [50]. For example, in the mitochondria of eukaryotic cells, the FAD domain, acting as the core part of the catalytic activity of PRODH, accepts hydrogen atoms from the proline molecule to promote a dehydrogenation reaction of l-proline, and the l-proline is oxidized to Δ^1^-pyrroline-5-carboxylate (P5C). During this process, the FAD domain is reduced to flavin adenine dinucleotide (FADH₂), and the FADH₂ transfers electrons to the electron transport chain (ETC) to generate ATP, providing energy for the cell [33, 51]. For *T. cruzi* and *T. brucei*, the FAD domain of both TcPRODH and TbPRODH accepted a hydrogen atom from the l-proline molecule, and the l-proline was oxidized to TcP5C and TbP5C, respectively [43]. The FAD was reduced to FADH₂ to transfer two electrons (2e) to ETC to generate ATP, providing energy for the intracellular growth of *T. cruzi* and *T. brucei* [48, 52]. In the present study, three regions (15–93 aa, 209–371 aa, and 405–481 aa) within the FAD binding domain were identified in the TgPRODH protein, consistent with the number of FAD binding regions in TcPRODH (93–123 aa, 154–199 aa, and 314–560 aa) of *T. cruzi* and TbPRODH (93–123 aa, 154–199 aa, and 314–554 aa) of *T. brucei*, while only one region of this domain was detected in PutA (262–618 aa) of *E. coli*. Based on the high similarity of the FAD domain in TgPRODH to the FAD-binding domains of TcPRODH and TbPRODH, the present study further identified three arginine sites (Arg^303^, Arg^450^, and Arg^451^) and two glutamic acid sites (Glu^435^ and Glu^454^) in the TgPRODH protein. These two sites were also reported in the TcPRODH (Arg^431^, Arg^555^, Arg^556^, and Glu^559^) of *T. cruzi* and the TbPRODH (Arg^392^, Arg^525^, Arg^526^, and Glu^529^) of *T. brucei* [53]. Furthermore, three regions (15–93 aa, 209–371 aa, and 405–484 aa) within the FAD binding domain, three arginine sites (Arg^303^, Arg^450^, and Arg^451^), and two glutamic acid sites (Glu^435^ and Glu^454^) were all consistent with the NcPRODH. The FAD has been reported to approach and bind to the specific sites of arginine and glutamic acid in the proline dehydrogenase. Through electrostatic interaction, the negatively charged region on FAD could be attracted by the positive charge of arginine, while the positively charged region on FAD could be attracted by the negative charge of glutamic acid [54]. In addition, the oxygen atom on the carboxyl group of glutamic acid has been found to combine with the hydrogen atom on the FAD domain to form a hydrogen bond to stabilize the binding of FAD to the proline dehydrogenase [55]. These findings suggested that the *Tgprodh* gene would play an important role for energy supply in the mitochondria of *T. gondii*.

Mitochondrial dysfunction caused by dysregulation of the proline dehydrogenase has been reported in model organisms and protozoan parasites. For example, the mutation of proline dehydrogenase 1 (*Atprodh1*) could not deliver electrons to the ETC during the proline oxidation process, resulting in mitochondrial respiratory disorders in *A. thaliana* [56], and silencing of the *Atprodh1* gene resulted in a decrease in the level of ROS produced by the mitochondria in *A. thaliana* [36]. Deletion of the proline dehydrogenase gene resulted in a significant swelling of the mitochondria in *Drosophila* and decreased the production of superoxide/H_2_O_2_ by the mitochondria in *Drosophila* [57, 58]. In protozoan parasites, overexpression of the *Tcprodh* gene increased the synthesis of ATP and the production of H_2_O_2_ in mitochondria by regulating the proline catabolism in *T. cruzi* [35]. However, RNA interference of the *Tbprodh* gene disrupted the proline catabolism in mitochondria of *T. brucei* and led to a decrease in ATP production by mitochondria in the absence of glucose [52]. The present study found that the absence of the *Tgprodh* gene induced a reduction in the density of mitochondrial cristae and an expansion of their morphology. In addition, deletion of the *Tgprodh* gene caused severe damage to mitochondrial function in *T. gondii*, including the reduction in ROS and ATP production, the decrease in membrane potential and mtDNA copy numbers, and downregulation of gene expression for maintaining mitochondrial integrity. Furthermore, the mitochondrial morphology (typical lasso-shaped) and the levels of ROS, ATP, mtDNA copy numbers, and the expression of genes for maintaining mitochondrial integrity in the complemented strain of the *Tgprodh* gene were restored. These findings indicate that the *Tgprodh* gene is crucial for maintaining mitochondrial function and also verify our hypothesis that the *Tgprodh* gene plays an important role for energy supply in the mitochondria of *T. gondii*.

The proline metabolism has been reported to occur in mitochondria of a great number of organisms, and the proline dehydrogenase is the first rate-limiting enzyme in the regulation of the proline metabolism [59]. Previous studies reported that deletion of the proline dehydrogenase gene resulted in abnormal proline metabolism in model organisms. For example, mutation of the *Osprodh* gene led to the accumulation of proline to reduce H_2_O_2_ accumulation and then oxidative stress occurred in rice [42], and knockout of the *prodh* gene in mouse hearts induced metabolic disorders of cardiomyocytes through increasing oxidative stress [60]. Knockout of the *prodh* gene resulted in reprograming amino acid metabolism, tricarboxylic acid cycle, urea cycle, and pentose phosphate pathway in MCF-7 breast cancer cells [61]. In retinal pigment epithelium (RPE) cells, mutation of the *prodh* gene caused substantial accumulation of proline in the liver, plasma, retina, and retinal pigment epithelium/choroid (RPE/Cho) [62]. In the present study, deletion of the *Tgprodh* gene resulted in the accumulation of proline and a significant reduction in the contents of glutamate and glutamine. In addition, the present study predicted that *T. gondii* encoded proline catabolic enzymes (TgP5CDH, TgGS, TgSSADH, TgPD-HE1β, and Tg2OG-Fe(II)_oxy) and proline synthase enzymes (TgPYCR1, TgPYCR2, TgOAT, TgG5K, and Tgγ-GPR). Notably, in the knockout strain of the *Tgprodh* gene, the mRNA levels of *Tgp5cdh*, *Tggs*, *Tgssadh*, *Tgpd-he1β*, and *Tg2OG-Fe(II)_oxy* were downregulated, while the mRNA levels of *Tgpycr1*, *Tgpycr2*, *Tgoat*, *Tgg5k*, and *Tgγ-gpr* were upregulated. However, the expression of these dysregulated genes were restored in the complementation strain of the *Tgprodh* gene. These results suggested that the *Tgprodh* gene would be involved in the proline metabolism in *T. gondii*.

## Conclusions

The present study identified a proline dehydrogenase (TgPRODH) encoded by *T. gondii*, and the *Tgprodh* gene was important for in vitro growth and in vivo pathogenicity. Further study revealed that the *Tgprodh* gene was crucial for the proline metabolism and mitochondrial function of the parasite. These findings suggest the importance of the proline metabolic pathway in *T. gondii* for its intracellular proliferation and pathogenicity. Our work also provides a potential therapeutic target against toxoplasmosis and a theoretical basis for the exploration on the mechanism of regulation of the energy metabolism in *T. gondii*.

## Supplementary Information

**Additional file 1: Table S1.** Information of primers in this study

**Additional file 2: Figure S1.** Amino acids of the PRODH proteins in different species were aligned.

**Additional file 3: Figure S2.** Phylogenetic analysis of the TgPRODH proteins in common protozoan were performed. Evolutionary analyses were conducted in MEGA X.

**Additional file 4: Figure S3** The endogenous labeling strain, knockout strain, and complemented strain of the *Tgprodh* gene in *T. gondii* were constructed by using CRISPR–Cas9 technology. **a** Schematic of the CRISPR–Cas9 strategy used for constructing the endogenous labeling strain of the *Tgprodh* gene in *T. gondii.* **b** The endogenous labeling strain of the *Tgprodh* gene in *T. gondii* identified by PCR. PCR1 confirmed the homologous recombination between the 5′ flanking arm of the *Tgprodh* gene and 10 × HA. PCR2 confirmed the homologous recombination between the 3′ flanking arm of the *Tgprodh* gene and 10 × HA. **c** Schematic of the CRISPR–Cas9 strategy used for constructing the knockout strain of the *Tgprodh* gene in *T. gondii.* **d** The knockout strain of the *Tgprodh* gene in *T. gondii* identified by PCR. PCR3 confirmed that the gRNA site at the *Tgprodh* gene locus cleaved. PCR4 confirmed the knockout of the *Tgprodh* gene locus. PCR5 confirmed the DHFR* successfully inserted into the cleavage site. **e** Schematic of the CRISPR–Cas9 strategy used for constructing the complemented strain of the *Tgprodh* gene in *T. gondii.* **f** The complemented strain of the *Tgprodh* gene in *T. gondii* identified by PCR. PCR6 confirmed knockout of the *Tguprt* gene locus. PCR7 confirmed the *Tguprt* gene locus replaced by the coding region of the *Tgprodh* gene. PCR8 confirmed the DHFR* successfully inserted into the cleavage site.

**Additional file 5: Figure S4.** Western blot and immunofluorescence analysis of the *Tgprodh*-HA, RHΔ*prodh-*HA and RHiΔ*prodh-*HA strains were performed. **a** Identification of *Tgprodh*-HA, RHΔ*prodh-*HA and RHiΔ*prodh-*HA strains by using Western blot. **b** Identification of *Tgprodh*-HA, RHΔ*prodh-*HA and RHiΔ*prodh-*HA strains by using immunofluorescence.

**Additional file 6: Figure S5.** The invasion and egress rate of RH, RHΔ*prodh*, and RHiΔ*prodh* strains in vitro were detected. **a** The intracellular invasion differences of three strains at 24 hpi. **b** The intracellular invasion differences of three strains analyzed by using immunofluorescence. **c** The intracellular egress differences of three strains at 48 hpi. **d** The intracellular egress differences of three strains analyzed by using immunofluorescence. Three independent experiments were conducted, and the differences were analyzed by Student’s *t*-test.

**Additional file 7: Figure S6.** Subcellular localization of TgPRODH were shown. The *Tgprodh*-HA strain was used as the experimental group, and the RH*ku80* strain was used as the control group. The scale bar is 20 μm.

## Data Availability

Data supporting the main conclusions of this study are included in the manuscript.

## Abbreviations

Vero: African green monkey kidney
DHFR: Dihydrofolate reductase
Δ: Gene deletion
iΔ: Gene complementation
PV: Parasitophorous vacuoles
L-Pro: l-proline
L-Glu: l-glutamic acid
L-Gln: l-glutamine
TEM: Transmission electron microscopy
DCFH-DA: 2′,7′-dichlorofluorescein diacetate
ROS: Reactive oxygen species
ATP: Adenosine triphosphate
∆Ψm: Mitochondrial membrane potential
mtDNA: Mitochondrial DNA
RT-qPCR: Reverse transcriptase quantitative polymerase chain reaction
SPF: Specific-pathogen-free
TgPRODH: Proline dehydrogenase of *T.* *gondii*
NcPRODH: Proline dehydrogenase of *N.* *caninum*
RHΔ*prodh*: Knockout strain of the *Tgprodh* gene
RHiΔ*prodh*: Complemented strain of the *Tgprodh* gene
*Tgprodh*-HA: Endogenous labeling strain of the *Tgprodh* gene
